# SAG-YOLO: A Lightweight Real-Time One-Day-Old Chick Gender Detection Method

**DOI:** 10.3390/s25071973

**Published:** 2025-03-21

**Authors:** Yulong Chang, Rongqian Sun, Zheng Yang, Shijun Li, Qiaohua Wang

**Affiliations:** 1 College of Engineering, Huazhong Agricultural University, Wuhan 430070, China; ylchang@webmail.hzau.edu.cn (Y.C.); srq@webmail.hzau.edu.cn (R.S.); yangzhengyzh@webmail.hzau.edu.cn (Z.Y.); 2 College of Animal Science and Technology, Huazhong Agricultural University, Wuhan 430070, China; lishijun@mail.hzau.edu.cn; 3 Ministry of Agriculture Key Laboratory of Agricultural Equipment in the Middle and Lower Reaches of the Yangtze River, Wuhan 430070, China

**Keywords:** chick gender, wing feather growth rate, machine vision, lightweight

## Abstract

Feather sexing, based on wing feather growth rate, is a widely used method for chick sex identification. However, it still relies on manual sorting, necessitating automation. This study proposes an improved SAG-YOLO method for chick sex detection. Firstly, the model reduces both parameter size and computational complexity by replacing the original feature extraction with the StarNet lightweight Backbone. Next, the Additive Convolutional Gated Linear Unit (Additive CGLU) module, incorporated in the Neck section, enhances multi-scale feature interaction, improving detail capture while maintaining efficiency. Furthermore, the Group Normalization Head (GN Head) decreases parameters and computational overhead while boosting generalization and detection efficiency. Experimental results demonstrate that SAG-YOLO achieves a precision (P) of 90.5%, recall (R) of 90.7%, and mean average precision (mAP) of 97.0%, outperforming YOLO v10n by 1.3%, 2.6%, and 1.5%, respectively. Model parameters and floating-point operations are reduced by 0.8633 M and 2.0 GFLOPs, with a 0.2 ms faster GPU inference speed. In video stream detection, the model achieves 100% accuracy for female chicks and 96.25% accuracy for male chicks, demonstrating strong performance under motion blur and feature fuzziness. The improved model exhibits robust generalization, providing a practical solution for the intelligent sex sorting of day-old chicks.

## 1. Introduction

Poultry meat and eggs are playing an increasingly important role in human dietary structure [1]. Among them, chickens constitute a significant portion of poultry consumption. In broiler production, male chicks are preferred due to their higher feed conversion efficiency and faster growth rates. In contrast, in layer production, female chicks are favored to maximize egg yield. As a result, more than 7 billion male chicks worldwide are culled annually via carbon dioxide suffocation or immersion [2,3]. Generally, female chicks reach sexual maturity earlier, produce more eggs, and consume less feed, while male chicks can be sold as premium poultry upon reaching maturity. Performing sex identification on day-old chicks enables precise management based on sex differences, which not only improves farming efficiency and economic benefits but also mitigates ethical concerns. Therefore, developing a rapid and non-invasive sex identification method for day-old chicks is an urgent necessity with significant practical implications.

Traditional chick sex identification methods primarily include vent sexing and molecular biology techniques. Vent sexing requires a high level of expertise and causes stress and potential injury to the chicks. Additionally, it is labor-intensive and subject to human error due to the operator’s subjective judgment [4,5,6,7]. Some researchers have applied well-established computer vision algorithms to chick sex identification by leveraging vent sexing experience to construct deep learning-based intelligent classification models [8,9,10]. However, these methods still rely on manually obtaining images through vent sexing and have yet to achieve full automation. Molecular-level techniques, such as DNA-based assays and PCR, provide high accuracy but are complex and time-consuming [11]. Otsuka M et al. utilized an endoscopic probe to observe chick gonads for sex determination, but this approach is invasive, time-consuming, and poses a risk of injury to the chicks [12]. Other studies have analyzed the acoustic features of chick vocalizations to determine sex [13,14,15], but this method is highly susceptible to environmental noise, requires independent data collection, and is challenging to implement in automated production settings. These conventional methods suffer from drawbacks such as potential harm to the chicks, inefficiency, and unsuitability for rapid and non-invasive detection.

With advancements in poultry breeding technology, sex identification based on the wing feather growth rate, a significantly sex-linked trait in chicks, has gained widespread adoption both domestically and internationally. This method identifies sex based on feather characteristics that differ between male and female chicks. Researchers have explored image-based approaches to replace traditional vent sexing, reducing chick stress and significantly lowering labor costs [16]. Latina M. A. et al. developed an image processing and support vector machine-based system for sex identification via wing feather growth rate, achieving an accuracy of 89% for male chicks and 90% for female chicks [17]. However, this method still requires manual positioning of the primary and covert wing feathers in front of the camera, introducing operational complexity and reliance on human intervention. Additionally, both two-stage and one-stage object detection algorithms, known for their balance between accuracy and speed, have demonstrated promising applications in poultry posture recognition and behavior analysis [18,19,20,21,22,23,24,25], providing a reference for wing feather growth rate-based sex identification of day-old chicks.

To address the issues of low efficiency, reliance on manual operation, and potential harm in existing chick sex identification methods, this study proposes a novel approach for the rapid and non-invasive identification of day-old chick sex based on wing feather growth rate. By integrating an improved lightweight neural network model, this method aims to enhance detection efficiency, reduce dependency on manual intervention, and minimize chick stress. The proposed approach offers a new avenue for precision management in poultry farming.

The main contributions of this study are as follows:

(1) A dedicated dataset for day-old chick sex identification was created, encompassing challenging scenarios such as mixed feather colors, indistinct feather characteristics due to similar hues, occlusion by fine down feathers, incomplete wing extension, and blurred features. This dataset provides a diverse and challenging testbed for algorithm optimization, facilitating more robust and generalized model performance.

(2) This study proposes three improvements to address the high computational complexity, insufficient multi-scale feature fusion, and large parameter volume of existing models. First, a StarNet lightweight backbone network is employed, which integrates spatial features through star operations. This approach increases the feature dimension without additional computational overhead, significantly reducing model complexity and parameter count, while enhancing feature extraction efficiency in complex scenes. Second, an Additive CGLU module is designed to improve the C2f structure. The additive similarity function and gating mechanism enhance multi-scale feature fusion capability, strengthening detail capture and model robustness, while maintaining the advantage of lightweight computation. Lastly, a GN Head is constructed, which reinforces feature correlation through group normalization and shared convolutional layers. This reduces the parameter volume while improving generalization ability, achieving a simultaneous optimization of both detection accuracy and efficiency.

(3) Ablation experiments were designed to assess the contribution of each improvement. Additionally, SAG-YOLO was compared with mainstream object detection models, including YOLO v5n, YOLO v7tiny, YOLO v8n, YOLO v10n, Faster R-CNN, and Cascade-RCNN, to evaluate its advantages in detecting wing feather growth rate in one-day-old chicks compared to existing models.

(4) In practical testing, when the chicks spread their wings, the wings that are not fully extended result in indistinct differences between the primary feathers and covert feathers of male and female chicks. This causes a decrease in recognition accuracy, leading to false positives or missed detections. To address this issue, a gender-detection sequence method was designed, based on the detection results of each frame of the image, by calculating ratios to determine the gender. The method was evaluated for its practical application performance of SAG-YOLO through testing and statistical analysis of detection results deployed on low-cost devices.

## 2. Materials and Methods

### 2.1. Dataset

#### 2.1.1. Experimental Samples

**Figure 1:**
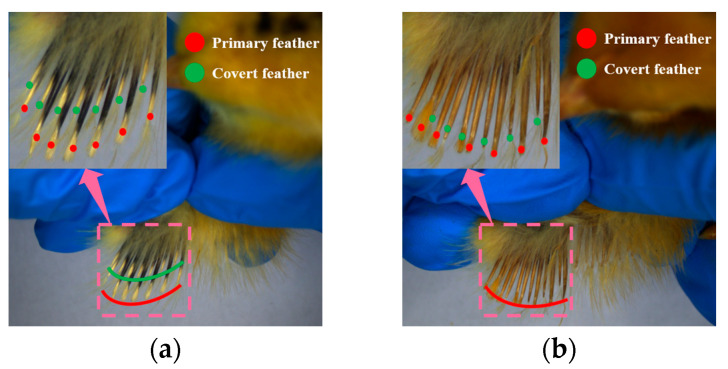
Mechanism of chick sex-sorting based on wing feather growth rate. (**a**) Female chick; (**b**) Male chick.

This study focuses on the Jianghan chicken breed selected by a poultry breeding company, using one-day-old chicks as the research subjects. Gender can be differentiated based on the wing feather growth rate, as female chicks have longer primary feathers near the wing tips compared to the covert feathers, whereas male chicks have primary and covert feathers of approximately equal length, as shown in Figure 1. Female Jianghan chicks are characterized by the early onset of egg production, high egg yield, and low feed consumption, while male chicks can be sold as high-quality chickens after reaching adulthood. The fine management of the flock based on gender differences can significantly improve breeding efficiency and economic productivity. Therefore, gender identification of one-day-old Jianghan chicks holds practical significance and provides a method for sex determination based on wing feather growth rate in one-day-old chicks. In this experiment, 201 female chicks and 188 male chicks were selected, with their gender determined by experienced workers from the poultry breeding company.

#### 2.1.2. Dataset Preparation

**Figure 2:**
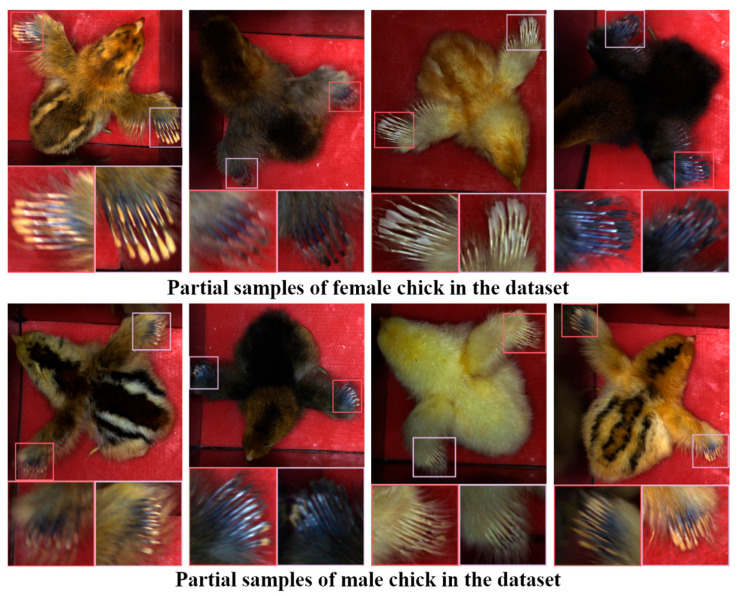
Part of dataset.

The video acquisition device used is the U3 3890 CP camera, manufactured by IDS GmbH in Germany, with an image resolution of 4000 × 3000. A total of 389 video segments were collected, capturing chicks in various postures. The samples exhibit considerable variation in individual color and feather growth conditions, making them relatively representative. As shown in Figure 2, both male and female chicks exhibit mixed feather colors, with black chicks having similar feather colors, making the features harder to distinguish. Light-colored chicks have dense down feathers, which obscure the areas of interest. Additionally, incomplete wing spreads or blurred features further increase the difficulty of recognition. This dataset reflects the practical challenges in recognition and provides diverse testing scenarios for algorithm optimization.

To avoid data leakage caused by adjacent frames being assigned to different sets due to frame extraction, the dataset was divided based on individual chicks. The data were split into a training set and a validation set using an 8:2 ratio with random stratified sampling. Frame extraction resulted in 5332 raw images, with 4209 images used for training and 1103 images used for testing. The primary wing feathers and coverts of the chicks were annotated using LabelImg, applying minimum bounding rectangles to reduce background interference. The chicks were labeled by sex as female and male. The details of the dataset split are shown in Table 1.

### 2.2. SAG-YOLO

YOLO v10 is an improved version of the YOLO series developed by the Tsinghua University team. It adopts a dual-label assignment strategy to achieve training without Non-Maximum Suppression (NMS), effectively reducing post-processing complexity and improving computational efficiency [26]. The model consists of three main components: the Backbone, Neck, and Head. The Backbone utilizes Cross Stage Partial Network (CSPNet) for feature extraction; the Neck integrates extracted features using a Path Aggregation Network (PAN); and the Head employs a one-to-many and one-to-one dual-label assignment strategy, eliminating the dependence on NMS. Additionally, the model incorporates PSA and C2fCIB modules to enhance performance with minimal computational cost. YOLO v10n is the smallest variant in the YOLO v10 series, and this study further optimizes it to better meet lightweight detection requirements.

**Figure 3:**
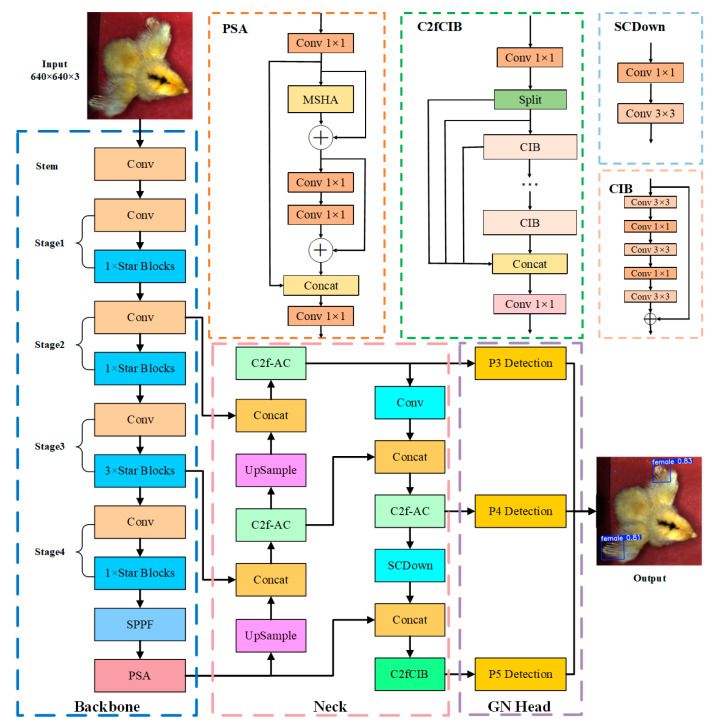
SAG-YOLO model architecture.

The Backbone serves as the model’s feature extraction module, responsible for capturing multi-level features from input images and passing them to the detection head for object recognition and localization. A lightweight and efficient Backbone is crucial for rapidly and accurately capturing key image features, significantly impacting overall network performance. This study replaces the YOLO v10n Backbone with StarNet, a lightweight backbone network based on star operations. StarNet effectively extracts distinguishing wing feather growth rate characteristics of day-old chicks in complex scenes while significantly reducing computational complexity and parameter count, making the model more suitable for resource-constrained environments. The Neck is responsible for multi-scale feature fusion and transmission. To enhance multi-scale feature fusion, this study introduces the Additive CGLU module to improve the C2f structure. This enhancement significantly strengthens multi-scale feature fusion capabilities and improves the capture of fine-grained features. As a result, the model demonstrates superior feature perception and robustness in challenging scenarios such as partially extended wings, blurred features, and complex backgrounds, ensuring better performance in chick sex identification. Additionally, the lightweight design of the Additive CGLU module maintains high computational efficiency while improving feature fusion, achieving an optimal balance between feature enhancement and detection efficiency. For the Head, this study designs a GN Head. The shared convolutional layers further enhance the connection between feature extraction and object detection, allowing the model to learn generalized features more effectively from the Neck output. The GN Head also leverages multi-scale feature maps for classification and regression tasks, reducing parameter count and computational complexity. This study introduces SAG-YOLO, an efficient and lightweight object detection model that reduces parameters and computational overhead while maintaining accuracy. The architecture of SAG-YOLO is illustrated in Figure 3, where C2f-AC represents the Additive CGLU-enhanced C2f module.

#### 2.2.1. Lightweight Feature Extraction Network: StarNet

Star operations represent an emerging learning paradigm that integrates different subspace features through element-wise multiplication, demonstrating exceptional performance and efficiency in computer vision tasks. Compared to traditional aggregation methods, star operations significantly expand feature dimensions without additional computational cost. When applied to neural networks and stacked across multiple layers, these operations enable exponential growth of feature dimensions within a compact feature space.

In a single-layer network, the star operation can be expressed as $\omega_{1}^{T}x*\omega_{2}^{T}x$, where element-wise multiplication fuses feature from two linear transformations. This formulation describes the feature dimension expansion process under a single-output channel transformation and single-element input scenario. The mathematical definition is given by $\omega_{1},\omega_{2},x\in R^{(d+1)\times 1}$, where d represents the number of input channels.

Under this condition, the output of a single-layer network is

(1) $$O_{1}=\omega_{1}^{T}x*\omega_{2}^{T}x=\sum_{i=1}^{d+1}\sum_{j=1}^{d+1}\omega_{\left(1,1\right)}^{i}{\omega_{\left(1,2\right)}^{j}x}^{i}x^{j}\;\;\in R^{(\frac{d}{\sqrt{2}}{)}^{2^{1}}}$$

For an l layer network, the output is formulated as

(2) $$O_{l}=W_{l,1}^{T}O_{l-1}*W_{l,2}^{T}O_{l-1}\;\;\in R^{(\frac{d}{\sqrt{2}}{)}^{2^{l}}}$$

According to Equation (1), the star operation expands into $\frac{\left(d+1)(d+2\right)}{2}$ independent components, each exhibiting a nonlinear relationship with input x, indicating that these components correspond to independent and implicit dimensions. Consequently, while the star operation is applied in a d-dimensional space, it effectively represents the feature distribution in an aaa-dimensional implicit feature space. As shown in Equation (2), stacking l layers allows the network to implicitly obtain a feature space belonging to $R^{(\frac{d}{\sqrt{2}}{)}^{2^{l}}}$. Therefore, by stacking multiple layers, star operations achieve an exponential expansion of the implicit feature dimension without introducing additional computational cost.

**Figure 4:**
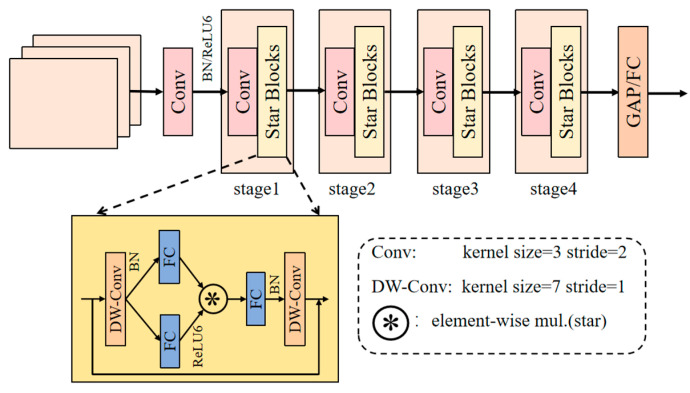
StarNet network architecture.

StarNet network structure [27] is shown in Figure 4. It consists of a STEM layer and four stages of convolution and feature transformation modules. The STEM layer uses a 3 × 3 convolution to expand the number of input channels from three to the initial channel count while reducing the image size. Each stage begins with a downsampling layer, progressively expanding the number of channels to higher dimensions while halving the image size, followed by multiple Star Block modules for feature extraction and fusion. Each Star Block module performs feature transformation using depthwise separable convolution and 1 × 1 convolution and maps input features to a highly nonlinear feature space through star operations. Finally, global average pooling and a fully connected layer are applied, utilizing the feature maps for subsequent classification and detection tasks. In this study, the StarNet network replaces the YOLO v10n Backbone, with an initial channel count set to 16, and the number of Star Block modules in the four stages is 1, 1, 3, and 1, respectively.

#### 2.2.2. Efficient Feature Fusion Module: Additive CGLU

**Figure 5:**
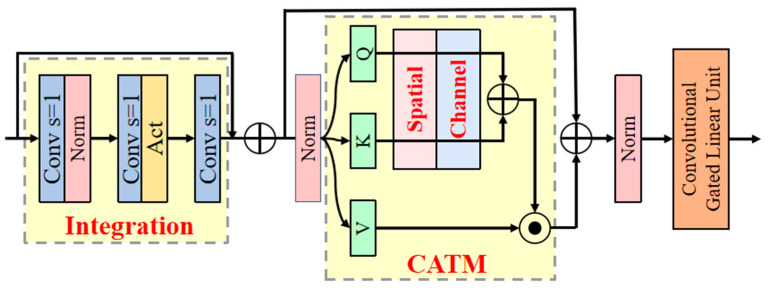
Additive CGLU module architecture.

This study designs the Additive CGLU module to replace the Bottleneck in the C2f module of the YOLO v10 Neck. It consists of three parts: Integration, Convolutional Additive Token Mixers (CATM), and CGLU, with its structure shown in Figure 5.

Integration is used to effectively extract and integrate image features. This module first adjusts the input feature channels to an intermediate dimension using a 1 × 1 convolution, reducing computational complexity and aggregating channel information. Then, depthwise separable convolutions based on channels are used to extract rich feature information in the local space, thereby effectively capturing local contextual relationships. Next, a nonlinear characteristic is introduced using an activation function to enhance the feature representation capability. Finally, a 1 × 1 convolution restores the output to the original number of channels. This part has a simple structure with low computational overhead, making it suitable for embedding into lightweight models to enhance feature representation capability. Moreover, Integration independently learns the local information of each channel in the feature space, enhancing the model’s ability to perceive target details and providing rich feature representations for downstream tasks.

(3) $$O_{Inte.}=Conv_{1\times 1}\left(ReLU\left(DepthConv_{3\times 3}\left(Norm\left(Conv_{1\times 1}\left(x\right)\right)\right)\right)\right)$$

CATM adopts a new interaction form based on spatial and channel attention to obtain global context information. In CATM, the similarity function is defined as the sum of the context scores of $Q\in R^{N\times D}$ and $K\in R^{N\times D}$:

(4) $$Sim\left(Q,K\right)=\Phi \left(Q\right)+\Phi \left(K\right)s.t.\Phi \left(Q\right)=C\left(S\left(Q\right)\right)$$

where Query, Key, and Value are obtained through independent linear transformations, such as $Q=W_{q}x,K=W_{k}x,V=W_{v}x$, with Φ(⋅) used for the context mapping function that includes the necessary information interactions. This generalized advantage is not limited to manually designed contexts but can also be achieved via convolution operations. Φ(⋅) specifically refers to channel attention $C(\cdot )\in R^{N\times D}$ and spatial attention $S(\cdot )\in R^{N\times D}$, which are based on depthwise convolutions and the Sigmoid activation function. Thus, the output of CATM can be represented as

(5) $$O_{CATM}=\Gamma \left(\Phi \left(Q\right)+\Phi \left(K\right)\right)\cdot V$$

where $\Gamma (\cdot )\in R^{N\times D}$ is the linear transformation that integrates the contextual information.

The CATM module performs similarity extraction on both the Query and Key branches, preserving the original features and enhancing the representation capability, reducing information loss in the 2D space. The use of Sigmoid activation for attention extraction instead of normalization facilitates network parallelization and deployment on mobile devices.

**Figure 6:**
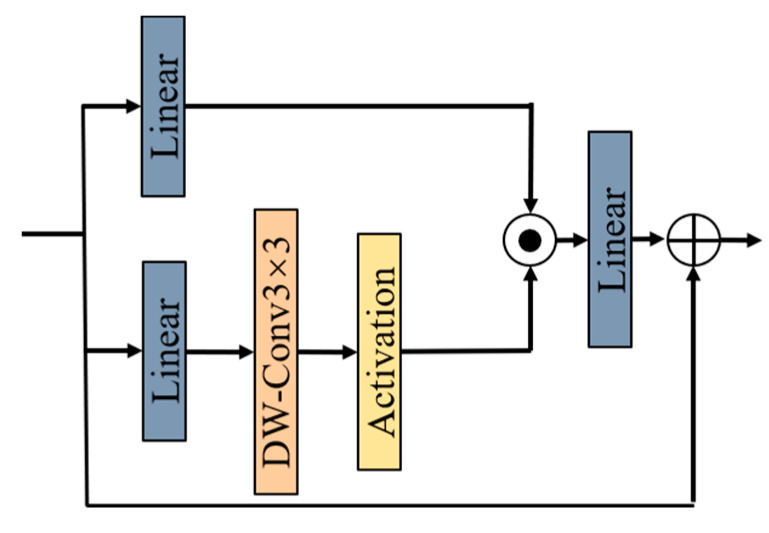
CGLU module architecture.

CGLU enables each token to obtain channel attention based on its neighboring image features, enhancing local modeling ability and model robustness. Its structure is shown in Figure 6. Specifically, CGLU first divides the input feature channels into two parts using a 1 × 1 convolution: one part undergoes depthwise convolution to capture spatial features, while the other part serves as the gating signal. The combination of 3 × 3 depthwise convolution and the GELU activation function satisfies the needs of fine-grained feature extraction and is friendly for backpropagation. Finally, a residual connection is used to add the input features back to the output, further stabilizing the gradient flow. CGLU achieves channel mixing attention with fewer computational resources, effectively enhancing the model’s robustness and making it more suitable for object detection tasks.

#### 2.2.3. High-Precision Lightweight Detection Head: GN Head

**Figure 7:**
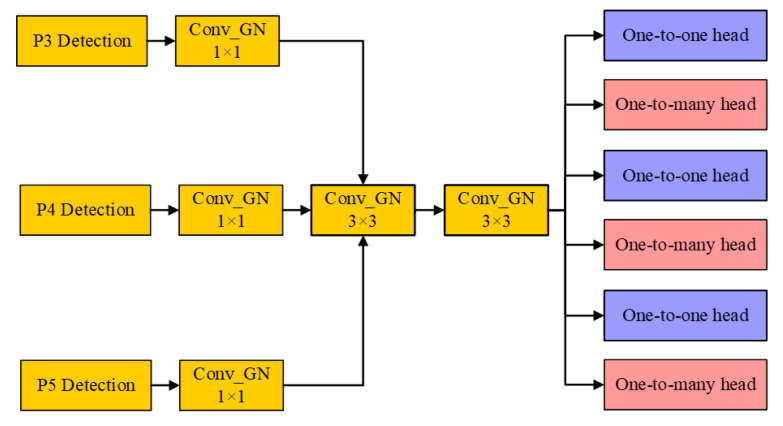
GN Head architecture.

The GN Head is an improved structure based on the YOLOv10n detection head, designed using shared convolutional layers. Its specific structure is shown in Figure 7. The output feature maps of the P3, P4, and P5 detection layers are divided into three groups. Each group of feature maps first undergoes convolution processing, followed by group normalization and SiLU activation function. Next, the feature maps enter the shared convolutional layer, which contains two convolution kernels that process the feature maps layer by layer. Because these two convolution kernels are shared across the detection layers, the same convolution operation can be reused, effectively reducing the number of model parameters and network complexity. This approach improves computational efficiency and reduces memory usage. The output feature maps from the shared convolutional layer are processed by regression and classification convolution layers to generate bounding box regression parameters and class prediction probabilities. The corresponding scale layers are applied to scale the features to ensure consistency across the target scales.

## 3. Results and Discussion

### 3.1. Experimental Environment Configuration and Model Training Parameters

The hardware configuration for model training is as follows: 128 GB of memory, an AMD Ryzen Threadripper 2920X CPU, and an NVIDIA RTX 2080Ti GPU with 12 GB of VRAM. The operating environment is Windows 10, Python version 3.8, with the PyTorch 1.9 framework and CUDA version 11.3. The input image size is 640 × 640 pixels, the batch size is 32, and the SGD optimizer is used with a momentum factor of 0.937, a weight decay factor of 0.0005, and an initial learning rate of 0.01. The model is trained for a total of 300 epochs.

### 3.2. Model Evaluation Metrics

The detection accuracy of the model is evaluated using P, R, and mAP. The complexity and computational cost of the deep learning model are measured Parameters, Giga Floating Point Of Operations (GFLOPs), and Size. The average inference time per image is used as the metric to evaluate the model’s speed. The calculation methods for P, R, and mAP are as follows:

(6) $$P=\frac{TP}{TP+FP}$$

(7) $$R=\frac{TP}{TP+FN}$$

(8) $$mAP=\frac{1}{N}\sum_{i=1}^{n}\int_{0}^{1}P(R)dR$$

In this context, True Positive (*TP*) refers to the number of samples predicted as positive and that actually are positive, False Positive (*FP*) refers to the number of samples predicted as positive but actually are negative, False Negative (*FN*) refers to the number of samples that are actually positive but predicted as negative, and *N* represents the total number of classes.

### 3.3. Ablation Study

An ablation study was designed to assess the contribution of each improvement point. Specifically, SAG-YOLO refers to the complete improved model, which incorporates StarNet as the Backbone, the C2f-Additive CGLU module as the improved Neck, and GN Head as the detection Head. AG-YOLO refers to the model with StarNet removed as the Backbone; SG-YOLO refers to the model with the C2f-Additive CGLU module removed from the Neck; and SA-YOLO refers to the model with GN Head removed as the detection Head. The results of the ablation study are shown in Table 2.

From Table 2, it can be observed that compared to SAG-YOLO, AG-YOLO has increased parameters by 0.4691 M, floating point operations by 1.4 GFLOPs, and memory usage by 0.9 MB. Additionally, its inference speed on the GPU is slower by 0.3 ms, and its P, R, and mAP have decreased by 0.1%, 1.1%, and 0.8%, respectively. This demonstrates that using the lightweight feature extraction network, StarNet, as the backbone for YOLO v10n significantly reduces computational load and network complexity while maintaining good feature extraction capabilities. When the C2f-Additive CGLU module is removed from SG-YOLO, compared to SAG-YOLO, its P, R, and mAP decrease by 3.0%, 3.6%, and 2.2%, respectively. Although this model is similar to the improved version in terms of parameters, floating point operations, memory usage, and inference speed, the results suggest that the C2f-Additive CGLU module effectively enhances the attention distribution of spatial and channel features. It retains low-level features while improving the model’s representational capability, thus, increasing detection accuracy. For SA-YOLO, which combines only StarNet and the C2f-Additive CGLU module, compared to SAG-YOLO, its P, R, and mAP decrease by 1.1%, 1.7%, and 1.1%, while parameters and floating point operations increase by 0.3223 M and 0.3 GFLOPs, respectively. This shows that the GN Head not only reduces model parameters and network complexity but also improves computational efficiency and reduces memory usage. Additionally, the shared convolutional layers in the end-to-end training mode further strengthen the connection between feature extraction and object detection, allowing the model to better learn general features and enhance generalization capability for different inputs. SAG-YOLO, which incorporates all the improved modules, achieves increases of 1.3%, 2.6%, and 1.5% in P, R, and mAP, respectively. It also has the lowest parameters at 1.4023 M and the lowest floating-point operations at 4.5 GFLOPs. Furthermore, its inference speed increases by 0.2 ms on a GPU with ample computing resources. SAG-YOLO demonstrates a comprehensive optimization of model size, accuracy, and inference speed, showcasing its strong potential in efficient detection scenarios.

**Figure 8:**
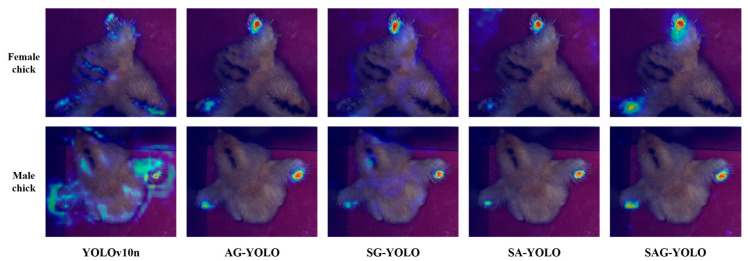
Grad-CAM visualization results.

To visualize the attention distribution of the models in the ablation study, Gradient-weighted Class Activation Mapping (Grad-CAM) was used. The visualization results are shown in Figure 8. In the image, the color gradient from blue to red indicates increasing levels of attention by the model. The base model’s attention focuses mainly on areas with no significant differences in fast and slow feathers. After introducing the C2f-Additive CGLU module, the model’s attention distribution on both spatial and channel features is effectively enhanced. Compared to the models without this module, YOLO v10n and SG-YOLO show significantly improved focus on the fast and slow feather regions. With the collaborative effects of the various improved modules, SAG-YOLO demonstrates significantly enhanced activation in the target areas, focusing more on the features with significant differences.

### 3.4. Comparison of Performance of Different Detection Models

To better evaluate the performance of the SAG-YOLO model, it was compared with Faster-RCNN, Cascade-RCNN, YOLO v5n, YOLO v7tiny, and YOLO v8n. The results are presented in Table 3.

From Table 3, it is evident that, as two-stage object detection models, Faster R-CNN and Cascade R-CNN did not achieve high precision in the chick feather recognition task, despite their fine detection capabilities. Additionally, they exhibited slower inference speeds and higher computational costs. In contrast, the SAG-YOLO model proposed in this study, which performs end-to-end prediction and detects objects across the entire image, is faster with lower computational complexity, making it more suitable for real-time detection applications, particularly in resource-constrained environments. Furthermore, when compared with YOLO series models, the SAG-YOLO model demonstrates significant advantages. In the chick gender detection task, YOLO v7tiny shows good inference performance, with a P of 87.7%, R of 90.1%, mAP of 94.7%, and an inference time of 6.2 milliseconds. However, the proposed SAG-YOLO model achieves a 2.3% improvement in mAP and significantly reduces the inference time to 1.9 milliseconds, more than three times faster than YOLO v7tiny. Additionally, the SAG-YOLO model reduces the number of parameters and floating-point operations by 76.7% and 65.4%, respectively, highlighting its significant advantages in resource usage and detection efficiency.

**Figure 9:**
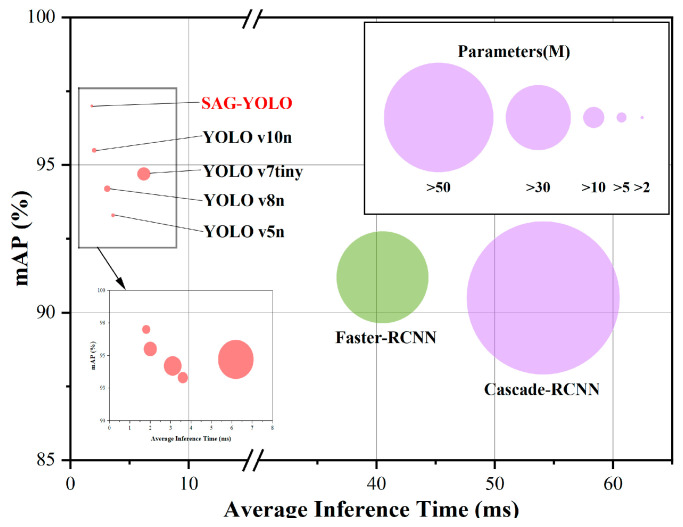
Average inference time and mAP of models with different parameter sizes.

The model’s parameter count, average inference time, and mAP are shown in Figure 9. The proposed SAG-YOLO model has achieved lightweight design and high efficiency, validating the effectiveness of the improvements.

**Figure 10:**
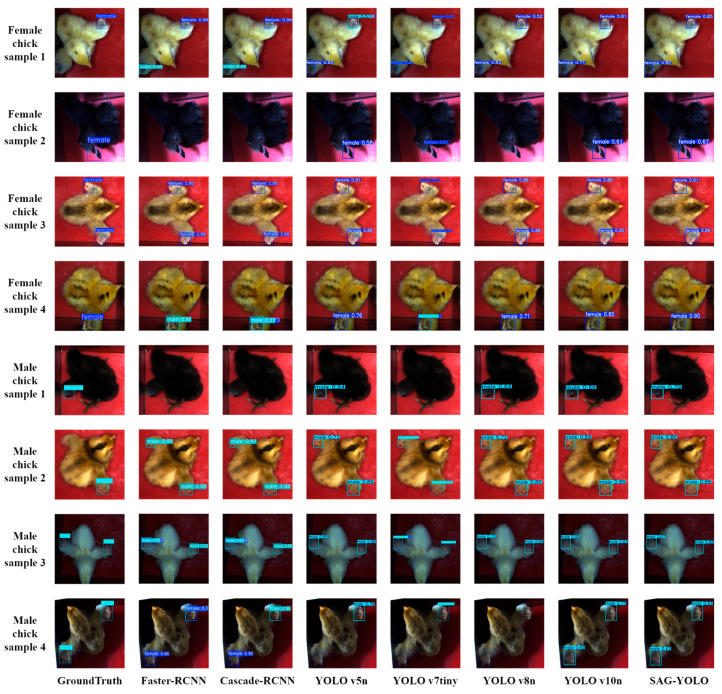
Detection results visualization.

The detection results are visualized in Figure 10, where the first column shows the Ground Truth, which provides accurate annotations of the target object positions and categories, and the remaining columns show the detection results of each model. For samples with distinct features, both YOLO series models and two-stage detection models can achieve efficient and accurate recognition for the gender sorting task. However, in complex scenarios, such as interference caused by the similarity in the color of black chicks, interference from the fluffy characteristics of light-colored chicks, and motion blur or incomplete wing extension of chick samples, other models tend to have issues like missed detections, false positives, or duplicate bounding boxes. In contrast, the SAG-YOLO model is capable of effectively learning the deep features of these challenging scenarios, demonstrating exceptional robustness and generalization ability. The SAG-YOLO model consistently achieves robust detection performance in challenging environments.

### 3.5. Practical Testing

To evaluate the model’s performance and generalization ability for chick sex detection under limited computational resources, 80 male and 80 female chicks of the same breed at one day old were selected for testing. Comparative experiments were conducted using the original models of Faster-RCNN, Cascade-RCNN, YOLO v5n, YOLO v7tiny, YOLO v8n, YOLO v10n, and the proposed SAG-YOLO model. The testing was performed on a laptop without a dedicated GPU, with inference carried out using the CPU, to validate the model’s lightweight performance.

During the process of the chicks spreading their wings, the camera captures several frames of images where the chick’s wings are not fully extended. At this stage, the differences between the male and female chicks’ primary feathers and covert feathers are not fully revealed, leading to a decrease in the model’s detection accuracy, which results in false positives or missed detections. However, when detecting individual chicks, no occlusion or interference from multiple targets occurs. In this study, only the detection results for the same target are considered. The accuracy of the model’s sex determination for one-day-old chicks is further evaluated based on the statistical proportion of correct classifications. During testing, the detection results of each frame are combined into a sequence of chick sex detections, where 0 represents a female chick and 1 represents a male chick. The final determination of the detection result is made based on the proportion (ratio) of the male/female classifications, which is greater than the threshold of 0.5. A portion of the chick detection sequences is shown in Table 4.

Based on the above statistical detection method, real-time video stream detection was performed on male and female chick samples. The test statistics for each model are shown in Table 5. The number of correctly identified samples, misjudged samples, and missed samples were separately counted. Accuracy is defined as the proportion of correctly identified samples out of the total number of detected samples. The average inference speed per frame is the average time taken by the model to infer the valid frames of the video stream during a single detection process.

As shown in Table 5, no missed detections occurred for female chicks in any of the target detection models. The two-stage object detection models, Faster-RCNN and Cascade-RCNN, each missed two and three male chick samples during detection, respectively. In contrast, all single-stage detection models missed only one male chick sample. In comparing the accuracy of correct and incorrect detections, except for the YOLO v5n model, which misidentified one female chick, all other models achieved 100% accurate detection for female chick samples. Faster-RCNN and Cascade-RCNN misidentified seven and six male chick samples, respectively. YOLO v5n achieved an accuracy of 93.75% for male chicks, while YOLO v5n, YOLO v7tiny, YOLO v8n, and YOLO v10n all achieved an accuracy of 92.50%. SAG-YOLO demonstrated the highest accuracy at 96.25%. Although the two-stage models, Faster-RCNN and Cascade-RCNN, performed similarly in terms of accuracy, their inference speeds were slower, reaching 4971.3 ms and 7949.1 ms, respectively, making them unsuitable for real-time applications. In comparison, SAG-YOLO, while maintaining the highest accuracy, achieved an average inference speed of 129.4 ms per frame. Overall, SAG-YOLO demonstrated the greatest potential for practical application in chick sex recognition tasks, owing to its outstanding accuracy and inference speed.

**Figure 11:**
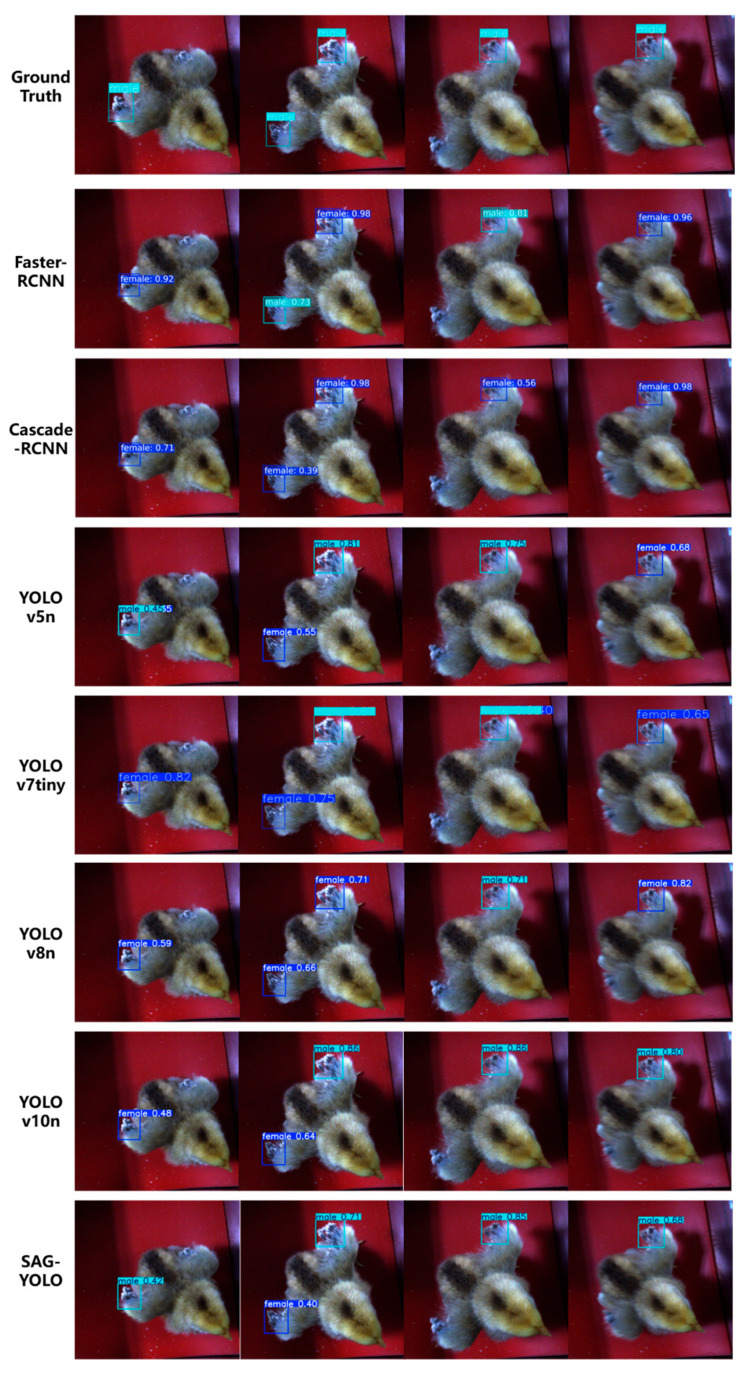
Visualization comparison of valid video frame testing.

As shown in Figure 11, when testing male chick samples with motion blur and difficult-to-distinguish features, the Faster-RCNN model made three feature misidentifications and two correct detections. Cascade-RCNN misidentified all five feature detections during the detection process. According to statistical analysis, two-stage detection models generally suffer from misidentification issues. The single-stage detection models YOLO v5n and YOLO v7tiny exhibited repeated bounding boxes around the same feature during detection, particularly for motion-blurred male chick samples. They failed to effectively suppress duplicate boxes, resulting in redundant outputs of multiple bounding boxes around the same target. The final sex determination was incorrect, showing the models’ limited adaptability in complex scenarios. YOLO v8n made four incorrect determinations and only one correct detection when identifying the wing feather growth rate features, indicating its inability to effectively extract features for inference in such scenarios. In contrast, both YOLO v10n and SAG-YOLO made correct determinations. SAG-YOLO, in particular, demonstrated superior feature recognition capabilities, successfully identifying the wing feather growth rate features of male chicks four times and providing accurate results, showing greater robustness and generalization ability.

### 3.6. Discussion

The SAG-YOLO model proposed in this study achieves a synergistic optimization of both accuracy and efficiency in the task of chick gender detection, compared to existing mainstream models. StarNet utilizes star operations to exponentially expand feature dimensions without incurring additional computational overhead. This design allows the model to efficiently extract key features even in complex scenarios, such as when feather colors are similar or when the wings are not fully spread. The Additive CGLU module strengthens the interaction of multi-scale features through an additive similarity function and gating mechanism. Ablation experiments show that removing this module results in a 2.2% drop in mAP, and Grad-CAM visualization results indicate a significant reduction in the focus on feature regions. This suggests that the module effectively mitigates the missed detection issue caused by information loss in traditional feature fusion by dynamically adjusting channel and spatial attention weights. The GN Head reduces parameter redundancy by sharing convolutional layers, while utilizing group normalization to enhance the model’s adaptability to different input distributions.

In real-time video stream testing, the SAG-YOLO model achieves detection accuracies of 100% and 96.25% for female and male chicks, respectively, with an inference speed of 129.4 ms per frame, significantly outperforming both two-stage models and YOLO series models. Its low computational complexity makes it suitable for low-cost devices, providing a feasible solution for automated sorting in poultry farms. However, future performance validation is needed to further assess its generalization capability under multi-variety and complex lighting conditions. Additionally, while the model performs excellently on GPUs, real-time performance in a pure CPU environment still requires optimization.

This study offers an efficient and lightweight solution for one-day-old chick gender detection, with a technical framework that can be extended to other poultry or agricultural object detection tasks. Future work will focus on constructing larger-scale, multi-scenario cross-variety datasets; exploring model quantization and deployment optimization on edge devices; and incorporating temporal information to further improve the robustness of video stream detection. With continuous improvement, this technology has the potential to drive the intelligent and sustainable development of the poultry industry, reducing resource waste and ethical concerns.

## 4. Conclusions

To address the challenge of automated chick sex detection, this study proposes the SAG-YOLO model. By replacing the backbone network with StarNet to reduce computational complexity, the model incorporates the Additive CGLU module to enhance multi-scale feature fusion, and the GN Head is designed to improve generalization and detection efficiency. The model achieves P of 90.5%, R of 90.7%, and mAP of 97.0%, representing improvements of 1.3%, 2.6%, and 1.5%, respectively, over the original model. Simultaneously, the number of parameters has been reduced by 0.8633 M, and the inference speed on the GPU has increased by 0.2 ms, achieving a comprehensive optimization in terms of lightweight design, accuracy, and inference speed. Furthermore, a model deployment method was designed, where the sex of the chick is determined based on the proportion of detection results in each frame’s sequence, effectively reducing misidentifications. The final test results demonstrate that the improved model achieves 100% detection accuracy for female chicks and 96.25% for male chicks. The overall accuracy significantly outperforms other detection models. With an average inference speed of 129.4 ms per frame, it still exhibits excellent feature recognition capabilities. The model’s low computational complexity allows for automated, non-destructive, one-day-old chick sex classification on low-cost devices, reducing labor costs, minimizing misidentification rates, and enhancing efficiency and economic benefits.

## Figures and Tables

**Table 1:** Dataset division.

Class	Total Samples	Training Set Samples	Validation Set Samples	Number of Training Images	Number of Validation Images
female	201	161	40	2339	587
male	188	150	38	1870	536
total	389	311	78	4209	1123

**Table 2:** Ablation experiment results.

Models	P/%	R/%	mAP/%	Parameters/M	GFLOPs	Size/MB	Average Inference Speed per Image on GPU/ms
YOLO v10n	89.2	88.1	95.5	2.2656	6.5	5.5	2.0
AG-YOLO	90.4	89.6	96.2	1.8714	5.9	4.1	2.1
SG-YOLO	87.5	87.1	94.8	1.4742	4.9	3.2	2.0
SA-YOLO	89.4	89.0	95.9	1.7246	4.8	4.8	1.9
SAG-YOLO	90.5	90.7	97.0	1.4023	4.5	3.2	1.8

**Table 3:** Comparison of results from different detection models.

Models	P/%	R/%	mAP/%	Parameters/M	GFLOPs	Size/MB	Average Inference Speed per Image on GPU/ms
Faster-RCNN	77.0	85.1	91.2	41.7530	72.1	162.0	40.5
Cascade-RCNN	78.8	82.0	90.5	69.3950	99.7	270.0	54.1
YOLO v5n	85.6	87.8	93.3	1.7619	4.1	3.9	3.6
YOLO v7tiny	87.7	90.1	94.7	6.0103	13.0	11.7	6.2
YOLO v8n	86.5	88.6	94.2	3.0060	8.1	6.1	3.1
SAG-YOLO	90.5	90.7	97.0	1.4023	4.5	3.2	1.8

**Table 4:** Chick gender classification sequence.

Samples	Number	Detection Sequence	Classification Result (Ratio > 0.5)
Male	1	1111111111	Male
Male	2	0101111111	Male
Male	3	1010111111	Male
Male	4	0111010000	Female (False Detection)
Female	5	0000000000	Female
Female	6	0010101010	Female
Female	7	0000100100	Female
Female	8	1010000000	Female

**Table 5:** Test results statistics.

Models	Test Samples		Correctly Identified Samples		Misjudged Samples		Missed Samples		Accuracy		Average Inference Speed per Frame/ms
	Male	Female	Male	Female	Male	Female	Male	Female	Male	Female	
Faster-RCNN	80	80	71	80	7	0	2	0	88.75%	100%	4971.3
Cascade-RCNN	80	80	71	80	6	0	3	0	88.75%	100%	7949.1
YOLO v5n	80	80	75	79	4	1	1	0	93.75%	98.75%	138.2
YOLO v7tiny	80	80	74	80	5	0	1	0	92.50%	100%	225.7
YOLO v8n	80	80	74	80	5	0	1	0	92.50%	100%	194.2
YOLO v10n	80	80	74	80	5	0	1	0	92.50%	100%	162.8
SAG-YOLO	80	80	77	80	2	0	1	0	96.25%	100%	129.4

## Data Availability

The data presented in this study are available on request from the corresponding author.
